# Mindfulness and Cardiometabolic Health During Pregnancy: An Integrative Review

**DOI:** 10.1007/s12671-024-02337-2

**Published:** 2024-03-28

**Authors:** Karen L. Lindsay, Yuqing Guo, Lauren E. Gyllenhammer

**Affiliations:** 1 Department of Pediatrics, UCI School of Medicine, University of California Irvine, 3800 W. Chapman Ave, Suite 2200, Orange, CA 92868, USA; 2 Susan Samueli Integrative Health Institute, University of California Irvine, Suite 4600, 856 Health Sciences Road, Irvine, CA 92697, USA; 3 Sue & Bill Gross School of Nursing, University of California Irvine, 854 Health Sciences Road, Irvine, CA 92697, USA

**Keywords:** Gestational weight gain, Glycemic control, Gestational diabetes, Hypertension, Mindfulness, Pregnancy

## Abstract

**Objectives:**

Cardiometabolic health during pregnancy has potential to influence long-term chronic disease risk for both mother and offspring. Mindfulness practices have been associated with improved cardiometabolic health in non-pregnant populations. The objective was to evaluate diverse studies that explored relationships between prenatal mindfulness and maternal cardiometabolic health.

**Method:**

An integrative review was conducted in January 2023 across five databases to identify and evaluate studies of diverse methodologies and data types. Quantitative studies that examined mindfulness as an intervention or exposure variable during pregnancy and reported any of the following outcomes were considered: gestational weight gain (GWG), blood glucose, insulin resistance, gestational diabetes, inflammation, blood pressure, hypertensive disorders of pregnancy. Qualitative studies were included if they evaluated knowledge, attitudes, or practices of mindfulness in relation to the above-mentioned outcomes during pregnancy.

**Results:**

Fifteen eligible studies were identified, and 4 received a “Good” quality rating (1/7 interventional, 1/5 observational, 2/2 qualitative). Qualitative studies revealed interest among pregnant women in mindfulness-based practices for managing GWG. Some beneficial effects of mindfulness interventions on maternal glucose tolerance and blood pressure were identified, but not for other cardiometabolic outcomes. Observational studies revealed null direct associations between maternal trait mindfulness and cardiometabolic parameters, but one study suggests potential for mindful eating to mitigate excess GWG and insulin resistance.

**Conclusions:**

There currently exists limited quality evidence for mindfulness practices to support prenatal cardiometabolic health. Further rigorous studies are required to understand whether prenatal mindfulness-based interventions, either alone or in combination with other lifestyle modalities, can benefit cardiometabolic health.

**Preregistration:**

This study is not preregistered.

The Developmental Origins of Health and Disease paradigm explains how suboptimal conditions or exposures in utero affect fetal development through alterations in cells, tissues, and organs, thus contributing to long-term disease risk in the offspring (Barker et al., 2002; Gluckman et al., 2007). In this regard, maternal cardiometabolic health during pregnancy, which encompasses gestational weight gain (GWG), glycemic control (including gestational diabetes mellitus [GDM]), blood pressure, and inflammation, plays a critical role in influencing child health outcomes (Brand et al., 2021; Han et al., 2021; Montazeri et al., 2018; Oken et al., 2009), while also influencing risk of adverse perinatal outcomes and long-term chronic disease risk for the mother (Farrar et al., 2016; Liu et al., 2016). Therefore, achieving optimal cardiometabolic health during pregnancy is an important public health consideration as it has potential to influence individuals’ health trajectories across generations.

In developed societies such as the United States, approximately 60% of pregnant women gain weight in excess of recommendations (Carmichael et al., 1997; Webb, 2008), 3–7% of pregnancies are affected by GDM (Catalano et al., 2012), and up to 16% are affected by hypertensive disorders (Ford, 2022). Excess GWG and GDM are both associated with increased odds of cesarean delivery and large for gestational age newborns (American College of Obstetrics and Gynecology, 2018a; Rogozińska et al., 2019), and additionally, GDM is associated with a 70% odds of future development of type 2 diabetes for the mother (American College of Obstetrics and Gynecology, 2018a).

Even in the absence of excess GWG or overt GDM diagnosis, maternal insulin resistance and hyperglycemia (collectively referred to as “glycemic control” in this paper) exert effects along a continuum and are associated in a graded, dose-dependent manner with increased risk of adverse maternal and child outcomes, including increased risk for childhood obesity and maternal risk for developing type 2 diabetes (Coustan et al., 2010; Durnwald et al., 2011; HAPO Study Cooperative Research Group, 2008, 2010a, 2010b; Vambergue et al., 2000; Walsh et al., 2011). Similarly, elevated blood pressure during pregnancy, even in the absence of preeclampsia, is associated with risk of preterm delivery and neonatal intensive care unit admission (Greenberg et al., 2021), and positively associated with offspring blood pressure (Jansen et al., 2019). Hypertensive disorders of pregnancy are also the leading cause of pregnancy-related mortality in the United States (Ford et al., 2022). Therefore, appropriate interventions and guidance on achieving a healthy cardiometabolic state during pregnancy (i.e., appropriate GWG, good glycemic control, blood pressure within normal ranges) are relevant for all pregnant women.

Existing interventions that aim to improve aspects of cardiometabolic health during pregnancy primarily focus on physical activity or dietary behaviors, but little attention is given to the role of psychological well-being in the pathophysiology of these conditions, or to the influence of poor psychological well-being on the very health behaviors that such intervention studies are attempting to alter (i.e., driving poor eating habits and sedentariness). Growing evidence supports that prenatal psychological well-being is associated with GWG, dysregulated glycemia, GDM, and hypertensive disorders of pregnancy (Caplan et al., 2021; Hartley et al., 2015; Horsch et al., 2016; Matthews et al., 2018; Shay et al., 2020). Observational studies have found that higher maternal distress (i.e., stress, depression, anxiety) may affect these markers of cardiometabolic health through direct pathways such as altered cortisol patterning (e.g., flattened cortisol decline and higher evening cortisol) (Gilles et al., 2018), lower insulin sensitivity index (Valsamakis et al., 2017), and reduced cardiac vagal control (i.e., lower high-frequency heart rate variability) (Rouleau et al., 2016), or through indirect pathways such as reduced dietary quality (Boutté et al., 2021; Lindsay et al., 2018) or physical activity levels (Sinclair et al., 2019).

Maternal stress and lifestyle factors may also interactively impact cardiometabolic health-related outcomes (Marques et al., 2015). For example, in a prospective longitudinal study, pregnant mothers’ higher negative emotions mitigated the beneficial effect of a Mediterranean diet on insulin resistance (Lindsay et al., 2020). In another prospective study, pre-existing anxiety disorders potentiated the effects of elevated maternal body mass index on development of hypertension during gestation (Winkel et al., 2015). Improving maternal psychological well-being holds potential to exert direct, beneficial effects on the underlying pathways involved in the pathophysiology of cardiometabolic outcomes in pregnancy. It also remains to be determined whether approaches to support maternal psychological well-being can indirectly promote improved cardiometabolic health by supporting engagement in and adherence to healthy lifestyle behaviors.

The prevalence of psychological distress (i.e., stress, depression, anxiety) during pregnancy has been reported to range from 5 to 20% in the US depending on the tools used for screening or diagnosis (Biaggi et al., 2016; Dunkel Schetter & Tanner, 2012). However, prior studies posit that even higher rates of low-grade chronic maternal distress may exist due to unique physiological, hormonal, socioeconomic, and interpersonal changes experienced during this time (Berthelot et al., 2020; Lin et al., 2020; Obrochta et al., 2020). Given the detrimental consequences of psychological distress on maternal outcomes, many studies have investigated the psychological impact of stress management interventions among pregnant women. Of these, mindfulness-based interventions have been frequently studied in recent years, as the benefits of mindfulness practice in non-pregnant populations have gained increasing attention. Mindfulness may be defined as the awareness that arises through paying attention, on purpose, in the present moment, non-judgmentally (Kabat-Zinn, 2003). Numerous reviews indicate that mindfulness-based interventions can reduce maternal perceived stress, depression, and anxiety during pregnancy (Dhillon et al., 2017; Hall et al., 2016; Lever Taylor et al., 2016; Lucena et al., 2020). Despite these favorable effects on psychological outcomes, there is a limited understanding of how mindfulness practice is associated with cardiometabolic outcomes during pregnancy. A number of systematic reviews of studies from non-pregnant populations indicate beneficial cardiometabolic effects of mindfulness-based interventions (Black & Slavich, 2016; Carriere et al., 2018; Dunn et al., 2018; Ni et al., 2021; Pascoe et al., 2017; Scott-Sheldon et al., 2020). However, there has been no published review to date assessing this evidence in the context of pregnancy.

A synthesis of current evidence from prenatal studies is required to understand whether and how these non-invasive, non-pharmacological approaches should be incorporated into future intervention studies with the goal of improving maternal health and pregnancy outcomes. We conducted a preliminary literature search that suggested a small number of diverse study types have been published on this topic in pregnancy cohorts (i.e., qualitative studies and quantitative studies with interventional and observational designs) although no pre-defined concept of gestational cardiometabolic health exists. Thus, a systematic review was deemed inadequate to comprehensively synthesize this literature given that it would require a narrow focus and would not accommodate heterogeneity in study designs and data types. Instead, an integrative review was considered the most appropriate approach as it serves to define concepts, review theories, synthesize evidence from diverse study methodologies, and analyze methodological issues to help guide future research (Whittemore & Knafl, 2005).

Therefore, this integrative review aimed to synthesize the existing evidence regarding the relationship between maternal mindfulness and cardiometabolic-related outcomes during pregnancy (i.e., GWG, GDM, glycemic control, insulin resistance, blood pressure, hypertensive disorders, inflammation). In addition to synthesizing quantitative data from existing observational and intervention studies, we also considered results from qualitative studies as these may be used to inform the design and implementation of future prenatal mindfulness interventions. This review also discussed ongoing research in this field and the limitations of existing work and suggested directions for future studies.

## Method

### Design

Given the infancy of the field of research on the physiological effects of mindfulness in pregnancy, an integrative literature review was selected as the review methodology as it facilitates a broad exploration of the topic across various study designs that incorporate diverse methodologies and generate various types of data (i.e., qualitative and quantitative), thereby including multiple perspectives. It also allows authors to define key concepts, analyze the existing evidence, and perform quality assessment of studies in the field, thus highlighting evidence gaps and needs for future studies to address. This approach differs from a scoping review which aims to identify the nature and extent of research evidence within a field when it is unclear what specific question should be addressed (Grant & Booth, 2009). Scoping reviews do not typically assess the quality of individual studies.

This integrative review was conducted following the guidelines of an established framework (Whittemore & Knafl, 2005) which includes the following five stages: (i) problem identification, (ii) literature search, (iii) data evaluation, (iv) data analysis, and (v) presentation of results and findings. After identifying the problem/research question, as outlined in the introduction section of this paper, key variables of interest were agreed upon to develop the search terms and define inclusion and exclusion criteria. An electronic search was conducted across several online databases to augment efficiency and to capture a broad scope of potentially relevant literature. The quality of each article selected for inclusion was systematically evaluated using validated quality assessment tools applicable to the individual study designs (see below under “Study Quality Assessment” for further details). Analysis was performed on included articles by abstracting and organizing data into pre-defined categories within tables for quantitative studies and by summarization of key themes identified from qualitative studies.

### Study Eligibility

Randomized controlled trials, quasi-experimental studies, observational studies (prospective or cross-sectional), and qualitative studies were all deemed eligible for this review. Conference abstracts, clinical guidelines, study protocols, and review papers were excluded, as were articles not published in the English language. Intervention studies were considered for eligibility if the intervention was either primarily based on mindfulness or incorporated some elements of mindfulness practice during pregnancy. Observational studies had to include a validated measure of mindfulness captured during pregnancy as an exposure variable in the analysis, and qualitative studies had to explore attitudes or beliefs surrounding mindfulness practice among pregnant women to be considered eligible. Yoga intervention studies were not deemed eligible unless they specifically described mindfulness techniques (e.g., mindful breathing or meditation) as a component of their intervention. This approach ensured that Westernized yoga practices that solely utilize the physical aspects of yoga as a form of exercise were not included. Interventions that exclusively targeted health behavior changes (e.g., dietary changes, exercise) without any concomitant mindfulness component were also excluded, as were studies conducted in the preconception or postpartum periods.

The pre-defined outcomes of interest for this review spanned four domains of cardiometabolic health in pregnancy: (i) gestational weight gain (absolute gain across pregnancy or rate of weight gain per week), (ii) glycemic control (fasting or post-prandial glucose, HbA1c, fasting insulin or measures of insulin sensitivity/resistance, GDM diagnosis), (iii) blood pressure (systolic or diastolic measures, hypertension, preeclampsia), and (iv) inflammation (inflammatory cytokines, C-reactive protein). For quantitative studies, objective or self-reported measures of at least one of the above prenatal cardiometabolic factors had to be assessed as a primary or secondary outcome variable to be considered eligible. Qualitative studies that considered at least one of these factors in relation to mindfulness practice as part of an interview or focus group in pregnant populations were considered eligible. Articles were excluded if they only reported maternal psychological outcomes, or if they only reported neonatal/birth outcomes without including any prenatal maternal cardiometabolic indices.

### Search Strategy and Study Selection

The published literature was searched using strategies created by consensus discussion between all authors and then final review by a librarian. These strategies were established using a combination of standardized terms and key words related to mindfulness, pregnancy, and cardiometabolic health (weight gain, glycemic control, blood pressure, inflammation); the complete search strategy is available as Supplementary Information. The following electronic databases were independently searched by one author (KLL) and a librarian in January 2023, without restriction by location or year: PubMed, CINAHL, Web of Science, Scopus, and PsycINFO. Articles returned within each database were independently screened by two authors (KLL, LEG) to identify those that potentially met eligibility criteria. Screening was initially conducted by title and abstract, and then by review of the full text of selected articles. The authors compared and discussed selected studies to resolve disagreements and reach consensus on the final article selection for in-depth review. Those selected were reviewed in their entirety by all authors and articles deemed not meeting inclusion criteria were discarded following reviewer consensus. Where required, corresponding authors of identified studies were contacted to obtain clarification on study methodology or results. The reference lists of selected studies and related review articles were also screened to identify further potential studies for inclusion.

### Data Extraction

Data extracted from the final selected studies included the study design, study location, sample size, participant demographics, details of the intervention/control group or exposure for quantitative studies, details of interview questions or discussion points for qualitative studies, and study results as pertaining to our pre-defined primary and secondary outcomes. Extracted data were summarized in tables and cross-checked for accuracy among reviewers, and discrepancies resolved by discussion.

### Study Quality Assessment

Appraisal of individual study quality was conducted independently by two authors (LEG, YG) using tailored tools according to study design. For quantitative studies, the quality assessment tools for controlled intervention studies and observational cohort/cross-sectional studies developed jointly by methodologists from the National Heart Lung and Blood Institute (NHLBI) and Research Triangle Institute International were used (U.S. Department of Health & Human Services, 2013). For qualitative studies, the Critical Appraisal Skills Programme (CASP) Qualitative checklist was used (Critical Appraisal Skills Programme, 2018). These tools were designed to assist reviewers in focusing on concepts that are key for critical appraisal of the internal validity of a study. Each tool includes items for evaluating potential flaws in study methods or implementation, and data analysis. Options of “yes,” “no,” or “cannot determine/not reported/not applicable” (or “Can’t Tell” on the CASP checklist) were selected in response to each item on each tool. For each item where “no” was selected, the potential risk of bias that could be introduced by that flaw in the study design or implementation was considered (this was true for both quantitative and qualitative studies). If the risk of bias was deemed to be high, the paper was deemed to have a critical flaw which resulted in an overall rating of poor quality. Outcomes of “cannot determine” and “not reported” for any item in the checklists for quantitative studies were also noted as representing potential flaws, but not necessarily critical flaws depending on the context in which they occurred and number of items in the checklist with such outcomes. Similarly, a rating of “Can’t Tell” for any item in the CASP checklist for qualitative studies was carefully considered a potential flaw in the research methodology and the impact that the uncertain information may have had on the study results was discussed among authors. A final rating of “good”, “fair,” or “poor” was assigned to each included study by each reviewing author, indicating either a low risk of bias, susceptible to some bias, or at high risk of bias, respectively. Individual appraisal results were discussed during face-to-face meetings such that the item ratings for each study were compared with rationale provided by each author. Where discrepancies arose, the rationale was deliberated among all authors using consensus decision-making to reach agreement on the final rating.

### Data Synthesis

Key features and primary results of included studies were organized into tables. A narrative synthesis of findings structured around geographical location, study design and primary research question, data collection methods and/or intervention, and results of the outcome measures pertaining to cardiometabolic health was provided.

## Results

### Study Selection

**Fig. 1:**
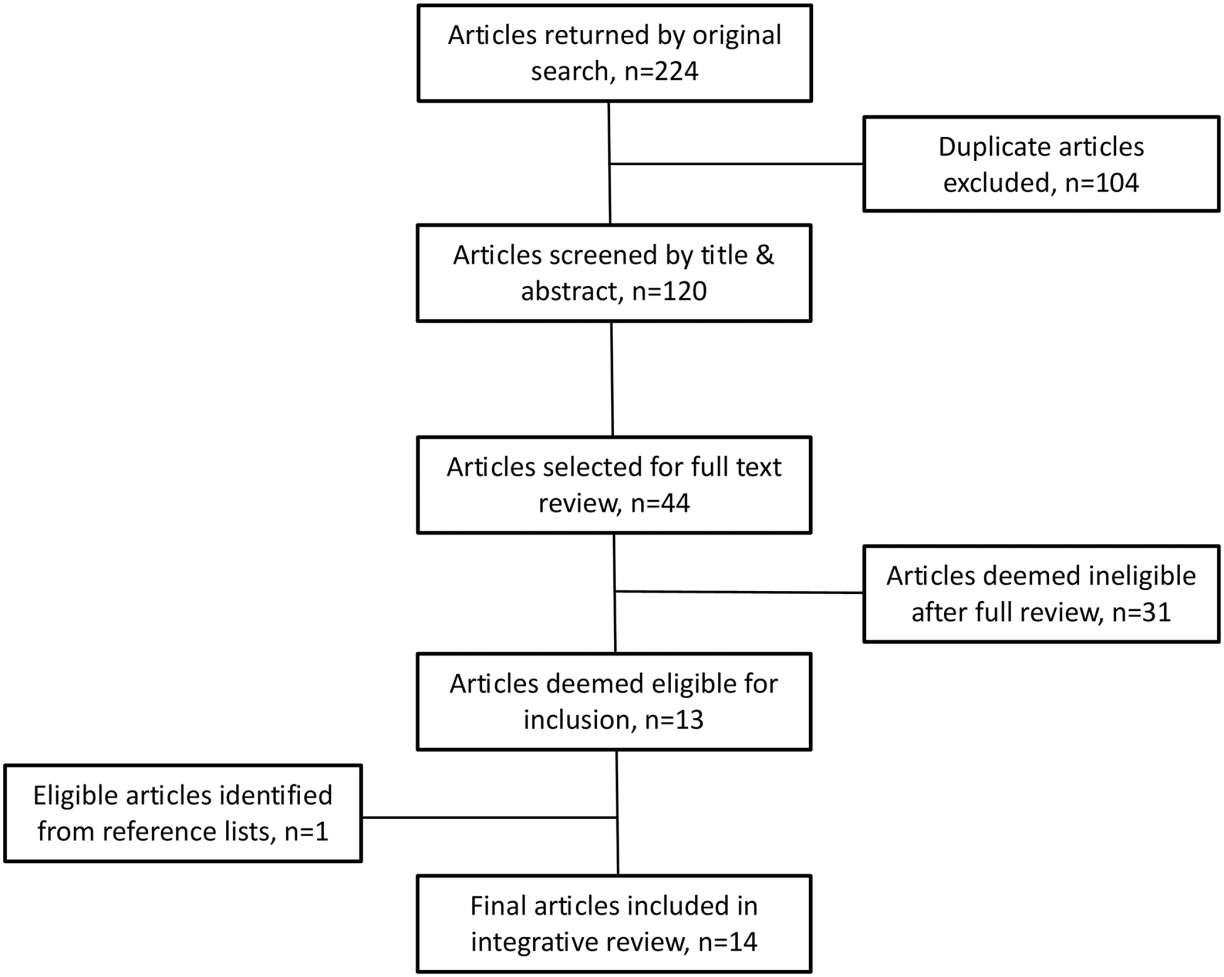
Flowchart of article search and selection process

An overview of the published article search and selection process is described in Fig. 1. The original search returned a total of 224 articles across all databases. This was reduced to 120 unique articles after removal of duplicates. Screening by title and abstract identified 44 potentially relevant articles with 90% agreement between authors. After full text screening of these articles and further discussion regarding potential eligibility, 31 were excluded by consensus: 3 study protocols, 7 review papers, 9 original research articles that did not incorporate a mindfulness exposure, 11 original research articles that did not report relevant maternal physiological outcomes, and 1 editorial comment. Literature cited in other review papers were screened for potential eligibility, but this did not identify any additional articles not already returned in our database search. One article returned by the database search was carefully considered for eligibility as the methodology only briefly mentioned mindfulness as a component of the behavioral intervention without further detail of the extent or content of the mindfulness practices employed (Opie et al., 2016). Contact with the corresponding author of that article confirmed that one-to-one counseling with study participants broadly incorporated mindful eating approaches, albeit in a non-standardized manner. The authors of this paper deemed that article to be relevant for inclusion based on the communication received. Review of reference lists identified one further article that was potentially eligible for inclusion (Redman et al., 2017); contact with the corresponding author of that paper confirmed that mindfulness techniques were systematically integrated into the diet and lifestyle intervention, and hence, the article was included. This brought the total number of included articles to 14; 12 quantitative studies, comprising of 7 interventions (Bublitz et al., 2023; Crovetto et al., 2021; Epel et al., 2019; Muthukrishnan et al., 2016; Opie et al., 2016; Redman et al., 2017; Youngwanichsetha et al., 2014) and 5 observational studies (Braeken et al., 2017; Headen et al., 2019; Lindsay et al., 2021; Matthews et al., 2018; Mennitto et al., 2021), as well as 2 qualitative studies (Green et al., 2021; Thomas et al., 2014).

### Study Quality Assessment

Detailed results of our quality assessment for each included study are presented in Supplementary Information Table S1. Among the intervention studies, only one was deemed to be of “Good” quality (Crovetto et al., 2021), and four were rated as “Fair” quality (Bublitz et al., 2023; Epel et al., 2019; Opie et al., 2016; Redman et al., 2017) and two of “Poor” quality (Muthukrishnan et al., 2016; Youngwanichsetha et al., 2014). The primary flaws identified that reduced study quality among the studies rated as “Fair” were failure to conduct a true intention-to-treat analysis (e.g., failure to account for missing outcome data) (Vorland et al., 2021; White et al., 2012), inadequate reporting of how missing data were handled, and baseline differences between intervention and control groups. Among the observational studies, only one was deemed to be of “Good” quality (Headen et al., 2019), two of “Fair” quality (Braeken et al., 2017; Lindsay et al., 2021), and two of “Poor” quality (Matthews et al., 2018; Mennitto et al., 2021). Various flaws were identified among the observational studies with a “Fair” and “Poor” quality designation, including risk of bias in the cohort selected for analysis or in outcome measures, and inadequate inclusion of potential confounding factors. Each of the two qualitative studies received a rating of “Good” quality.

### Description of Included Studies

The characteristics of included quantitative studies are described in Supplementary Information Table S2.

#### Intervention Studies

Two of the intervention studies had a quasi-experimental design, such that data for the control groups were obtained from a convenience sample of pregnant patients through electronic medical records (Opie et al., 2016) or the control arm comprised either those who refused participation in the intervention arm due to schedule conflicts (Epel et al., 2019). Five of the intervention studies were randomized controlled trials. Among these, two involved three study arms such that two alternative intervention groups were compared to one another as well as a control group, which reflected treatment as usual (Crovetto et al., 2021; Redman et al., 2017). Three studies targeted high-risk pregnancies including those with a history of hypertensive disorder, risk factors for small for gestational age birth, or with a diagnosis of GDM (Bublitz et al., 2023; Crovetto et al., 2021; Youngwanichsetha et al., 2014).

Four of the intervention studies delivered an intervention component that was centered around standardized or adapted versions of existing mindfulness programs, such as Mindfulness-Based Stress Reduction (MBSR) (Crovetto et al., 2021). One study delivered a non-standardized intervention that combined mindful eating didactic sessions with yoga practice (Youngwanichsetha et al., 2014). The other two intervention studies were centered around diet and lifestyle behavior change to support pregnancies with overweight and/or obesity and incorporated some mindful eating or mindful stress reduction guidance as minor components of the interventions (Opie et al., 2016; Redman et al., 2017). For the cardiometabolic outcome measures, one study focused solely on GWG (Altazan et al., 2019), a second study only focused on glycemic control (Youngwanichsetha et al., 2014), and two studies only focused on blood pressure–related outcomes (Bublitz et al., 2023; Muthukrishnan et al., 2016). The other three studies reported some combination of two of these outcome domains, but none reported on all three. Each of these cardiometabolic outcome measures was either objectively measured by the research team or abstracted from the medical record (e.g., diagnosis of GDM or preeclampsia). No study reported on inflammatory outcome measures.

#### Observational Studies

The included observational studies employed a mixture of study designs, including prospective cohort (Braeken et al., 2017; Lindsay et al., 2021), cross-sectional (Matthews et al., 2018; Mennitto et al., 2021), and a retrospective observational study (Headen et al., 2019) that conducted secondary data analysis from one of the intervention studies included in this review (Epel et al., 2019). None of the observational studies involved pregnancies with underlying medical complications. Four studies evaluated trait mindfulness of pregnant mothers using standardized, validated constructs (Braeken et al., 2017), and the final study evaluated how neighborhood typology of pregnant women moderated the effectiveness of a structured mindfulness intervention on measured health outcomes (Headen et al., 2019). Three studies reported objectively measured cardiometabolic outcomes (Braeken et al., 2017; Headen et al., 2019; Lindsay et al., 2021) while two relied on maternal self-reported outcomes (Matthews et al., 2018; Mennitto et al., 2021). Matthews et al. (2018) evaluated the relationship between trait mindfulness and self-reported GWG by trimester but did not specify in the methodology if those in second or third trimesters were asked to recall GWG in their earlier trimesters, or if they only reported total GWG up until the time of completing the survey.

#### Qualitative Studies

Of the two qualitative studies included in this review, one conducted focus groups with 59 low-income pregnant women with overweight or obesity to gain their perspectives on the relationship between stress, eating behavior, and weight gain in pregnancy and their interest in engaging in a prenatal mindfulness-based intervention to help support diet and achieve a healthy GWG (Thomas et al., 2014). The authors of that study subsequently published the paper describing the results of the Mindful Moms Training intervention, which is also included in this review (Epel et al., 2019). The second qualitative study conducted interviews with 13 healthy pregnant women who had recently completed participation in a 12-week prenatal yoga intervention (Green et al., 2021). That study sought to gain insight to mothers’ experiences of the yoga intervention and their perceived barriers and facilitators to engaging in prenatal yoga for the prevention of excess GWG. Interview questions included an exploration of mothers’ perceptions of mindfulness and how it relates to GWG. The parent intervention study from which this qualitative study was derived was not identified in the published literature at the time of conducting this review.

### Synthesis of Study Results

Table 1 summarizes the key results related to cardiometabolic outcomes for the included quantitative studies.

#### Intervention Studies

Of three studies reporting effects on GWG, only Redman et al. (2017) found a significantly lower proportion of excess GWG in both intervention arms versus control in their randomized controlled trial. However, the two quasi-experimental studies did not identify any significant benefit of the intervention, although Epel et al. (2019) reported a significantly higher proportion of GWG that fell below the recommended level according to BMI category compared to the control. Of note, the other quasi-experimental study was missing more than 50% of the GWG data for their control group (Opie et al., 2016).

For glycemic outcomes, no interventional effects were noted for GDM incidence, but there was benefit regarding maternal glucose concentrations. Specifically, GDM rates were not impacted by the MBSR intervention compared to control in the study by Crovetto et al. (2021), nor by the behavioral intervention incorporating a modest amount of mindful eating coaching in the quasi-experimental study by Opie et al. (2016), after adjusting for covariates. However, both Epel et al. (2019) and Youngwanichsetha et al. (2014) reported lower glucose values on glucose tolerance tests among their mindfulness intervention groups compared to control. Youngwanichsetha et al. (2014) additionally found modestly lower fasting glucose values in the intervention group versus control.

Results for mindfulness intervention effects on blood pressure were mixed across three studies, with two studies indicating some benefit, whereas one study showed no effect of intervention. Bublitz et al. (2023) reported significantly lower systolic and diastolic values (at rest) in the intervention group compared to control. While Muthukrishnan et al. (2016) reported no difference between groups in resting blood pressure values, they did demonstrate an improved blood pressure response to physical and psychological stress tests (i.e., a smaller increase in both systolic and diastolic blood pressure) compared to control. Crovetto et al. (2021) reported no effects of the mindfulness intervention on blood pressure, as well as no effects on rates of preeclampsia or gestational hypertension, compared to control.

#### Observational Studies

Two observational studies examined associations between self-reported mindfulness traits and GWG and reported some evidence that greater trait mindfulness associated with lower GWG across pregnancy. While Lindsay et al. (2021) reported no association between the total mindful eating score and rate of GWG per week in pregnant women with obesity, there was a relationship when examining subdomains of mindfulness. Specifically, there was a significant relationship with the distracted eating subscale from the mindful eating questionnaire, such that greater mindful eating practice in this domain associated with a lower rate of GWG. Matthews et al. (2018) reported that higher trait mindfulness scores were negatively associated with maternal self-reported GWG in the first trimester only. However, we note that the GWG data collection methods were incompletely described in that paper, which challenged interpretation of the results. In a secondary analysis, Headen et al. (2019) reported no moderating effect of participants’ neighborhood typology on the effect of a prenatal mindfulness intervention on rate of excess GWG.

Regarding glycemic-related outcomes, Lindsay et al. (2021) reported that higher mindful eating scores were significantly associated with lower insulin resistance in the third trimester of pregnancy, measured by the homeostasis model assessment of insulin resistance. Headen et al. (2019) identified a significant moderating effect of neighborhood typology on the effects of the Mindful Moms Training intervention on glucose tolerance in pregnancy, such that only those in the best-resourced and least-resourced neighborhoods were observed to have better glucose control following the intervention. Mennitto et al. (2021) reported incidence of GDM as the only glycemic-related outcome but found no association between trait mindfulness and its occurrence.

Only one observational study reported systolic and diastolic blood measurement outcomes but found no association between maternal trait mindfulness and either measure (Braeken et al., 2017). Similarly, trait mindfulness was not associated with incidence of gestational hypertension in the study by Mennitto et al. (2021).

#### Qualitative Studies

Themes identified by the focus groups conducted by Thomas et al. included “relationship between stress and eating” and “motivations for a stress reduction intervention during pregnancy” (Thomas et al., 2014). Most participants acknowledged a complex relationship between stress, emotions, and eating in their lives, recognizing tendencies for mindless eating under stress, and consequences for excess GWG. The participants also expressed enthusiasm about a mindfulness-based stress management intervention tailored to pregnancy that could help support healthy eating behaviors and healthy GWG. In particular, they were interested in a group intervention with other pregnant women that went beyond traditional dietary counseling by incorporating mindfulness and were optimistic that this type of intervention could help them cope with stress and address their concerns about GWG.

Green et al. (2021) identified 12 themes from their interviews with pregnant women who participated in a prenatal yoga intervention to help manage GWG. One of these themes, “prenatal yoga and weight,” was particularly related to mindfulness as participants reported that engaging in the yoga practice increased their sense of self-awareness and mindfulness in their daily lives, with beneficial effects on eating habits. For example, participants expressed that they became more mindful of what food they were putting into their body and how their dietary choices could affect the health of their baby and weight gain. Some also expressed that increasing levels of mindfulness helped them become aware of how much weight they were gaining and whether it was too much or too little.

## Discussion

This integrative review presents a detailed synthesis of currently available evidence linking mindfulness practice in pregnancy to cardiometabolic-related outcomes in the mother. With the increasing popularity of studies that involve mindfulness interventions in pregnancy (Lucena et al., 2020), it is important to understand if potential benefits may extend beyond psychological aspects of maternal health and support cardiometabolic health outcomes, especially given rising rates of maternal obesity, GDM, and hypertensive disorders of pregnancy. Growing evidence supports beneficial effects of mindfulness on weight management, glycemic, and blood pressure outcomes in non-pregnant populations (Black & Slavich, 2016; Carriere et al., 2018; Dunn et al., 2018; Ni et al., 2021; Pascoe et al., 2017; Scott-Sheldon et al., 2020). Although this integrative review found limited and mostly low-grade quantitative evidence to support benefits of mindfulness on cardiometabolic outcomes during pregnancy, the inclusion of high-quality qualitative evidence highlights promising attitudes among participants of mindfulness interventions and strengthens the premise to advance more rigorous research on this topic in prenatal populations. This review also highlights several methodological inadequacies across included studies, as well as heterogeneity among intervention studies regarding the type, intensity, and delivery of mindfulness-based approaches, which limits our ability to draw firm conclusions regarding the status of the evidence in this field. Notably, no study was identified that reported outcomes related to inflammatory markers during pregnancy, representing a missed opportunity to understand the underlying biological pathways that could link mindfulness practice to improvements in pregnancy outcomes.

### Prenatal Mindfulness and GWG

Among intervention studies that delivered some type of formal mindfulness training, only Epel et al. (2019) reported GWG as an outcome but found no benefits for reduced rate of excess GWG compared to control. However, their finding that a higher proportion of women in the intervention group had GWG below the recommended range for their BMI category (overweight and obese groups) deserves further attention. This may not necessarily represent an adverse outcome given that the National Academy of Medicine guidelines for GWG (Rasmussen & Yaktine, 2009) have been criticized for being too generous and that those with pre-pregnancy obesity may benefit from less GWG to offset the adverse effects that higher adiposity may exert on pregnancy and infant outcomes (Barbour, 2012; Robillard, 2023).

While Redman et al. (2017) reported a significantly lower rate of excess GWG among pregnant women with obesity in both healthy lifestyle intervention groups (in-person and remotely delivered) compared to control, we note that mindfulness techniques represented a minor component of those interventions compared to other lifestyle practices (i.e., nutrition counseling, exercise). Although we are unable to decipher which component of that broader lifestyle intervention may have exerted the observed effects, it is plausible that incorporating mindfulness approaches could enhance the effects of lifestyle behavior change counseling on maternal GWG. For example, in a weight loss randomized controlled trial in non-pregnant adults, a diet and exercise intervention combined with mindfulness training was found to significantly reduce reward-driven eating behavior compared to diet and exercise intervention alone, and the reduction in reward-based eating mediated the effect of the combined intervention arm on weight loss at the 12-month follow-up time point (Mason et al., 2016). Thus, further research is required to elucidate whether mindfulness strategies that support maternal psychological well-being in pregnancy can augment the effectiveness of traditional behavioral lifestyle interventions with respect to important cardiometabolic health outcomes.

Observational studies included in this review identified modest associations between maternal mindful eating behavior (Lindsay et al., 2021) or trait mindfulness (Matthews et al., 2018) and GWG, albeit the quality of this evidence was fair to poor. However, the two qualitative studies indicate that pregnant women are both interested in and perceived benefit from engaging in mindfulness practices to support healthy GWG (Green et al., 2021; Thomas et al., 2014). Importantly, Thomas et al. (2014) conducted their focus groups among a population of low-income, predominantly Hispanic pregnant women, who typically experience higher rates of excess GWG compared to non-Hispanic White women and therefore have potential to exert greater benefits from such interventions. Although Green et al. (2021) evaluated participant feedback on a prenatal yoga intervention rather than formal mindfulness training, participants of that study reported how the yoga practice increased their awareness of health practices and weight management during pregnancy and specifically identified a sense of greater mindfulness from which they perceived benefit. These qualitative studies, which both received a “Good” quality rating, can help inform future prenatal mindfulness interventions with respect to approaches used to elicit motivation and engagement among target participants.

### Mindfulness and Glycemic Control

Only one intervention study (Crovetto et al., 2021) and one observational study (Mennitto et al., 2021) included in this review reported the incidence of GDM in relation to either formal mindfulness training or measured trait mindfulness, respectively, and neither found any evidence for beneficial effects of mindfulness in this regard. We note that the study by Crovetto et al. was the only intervention study included in this review that received a “Good” quality rating, thus increasing credibility of the results reported. However, that study was not powered to assess the impact of mindfulness practice on the incidence of GDM, and therefore, we cannot conclude with certainty that there was a null effect. The quasi-experimental lifestyle intervention by Opie et al. (2016) reported a notably lower rate of GDM in the intervention group versus control, but this was only statistically significant before adjusting for confounding factors. However, it is not possible to attribute the observed differences in GDM rate between groups to increased maternal mindfulness given that the intervention group only received mindful eating advice in a non-systematic manner during nutrition consultations, rather than undergoing any type of formal mindfulness training or counseling on mindful eating. Furthermore, the study by Opie et al. (2016) received a “Fair” quality rating and the reliability of the results reported should be carefully considered.

Despite this lack of evidence with respect to GDM as a diagnostic outcome, our review did identify several studies that implicate potential beneficial relationships between increased maternal mindfulness and glycemia during pregnancy as measured on a continuous spectrum. Lower glucose concentrations in the intervention groups versus control following glucose tolerance tests in two of the intervention studies may indicate a role for formal mindfulness practices to help improve glucose-insulin homeostasis (Epel et al., 2019; Youngwanichsetha et al., 2014). However, we also must interpret these results with caution given that those studies received a “Fair” and “Poor” quality rating, respectively. Data from one observational study also implicates higher maternal mindful eating behavior to be associated with lower levels of insulin resistance in the third trimester (Lindsay et al., 2021), although that study also received a “Fair” quality rating and was not powered to detect the observed associations. Collectively, these findings hold clinical relevance given that moderately elevated maternal glucose concentrations, even in the absence of GDM, have been found to be positively associated with neonatal and child adiposity (HAPO Study Cooperative Research Group, 2009; Kaul et al., 2022). Also, higher levels of gestational insulin resistance, even among those receiving treatment for GDM, have been associated with greater risk for adverse pregnancy outcomes (Benhalima et al., 2019; Cosson et al., 2022).

Future well-designed and higher quality prenatal mindfulness intervention studies are clearly needed to see if these findings with respect to maternal glycemia can be replicated. However, it is recommended that such studies also consider the underlying influence of maternal demographic factors on the efficacy of such interventions, and how the interventions can be appropriately tailored to meet the needs of underserved populations. For example, Headen et al. (2019) demonstrated that neighborhood typology (classified according to wealth and resource access) moderated the effects of the Mindful Moms intervention on glycemic control following a glucose challenge test. Structural and social determinants of health are likely responsible for these disparate effects (American College of Obstetrics and Gynecology, 2018b) as exposure to poverty and having low access to resources, such as healthy food and healthcare, may adversely affect glycemia to the extent that a mindfulness intervention is unable to counteract. Furthermore, individuals living in poverty and with low resource access are more likely to experience heightened psychological stress and various competing demands for time (Bermúdez-Millán et al., 2011; Vaughan et al., 2023), which may challenge their ability to attend and sufficiently engage in supportive health services such as those offered by the Mindful Moms intervention. Interestingly, Headen et al. (2019) found that those from poor neighborhoods with moderate resource access experienced improved glucose tolerance following the intervention compared to the control arm, which may indicate that better neighborhood access to resources supports one’s ability to benefit from a prenatal mindfulness practice despite having a low-income level.

### Mindfulness and Blood Pressure

The limited available evidence to date describing relationships between prenatal mindfulness and maternal blood pressure shows mixed results. The largest intervention study included in our review, which also received the highest quality rating compared to other intervention studies, reported null effects of a MBSR program on rates of preeclampsia or gestational hypertension (Crovetto et al., 2021). However, we note that these represented secondary outcome measures in that study and that the primary outcome, rate of small for gestational age at birth, was significantly lower in the mindfulness intervention group compared to control. The apparent success of the intervention on reducing small for gestational age may have been achieved through biological mechanisms related to psychological stress reduction rather than any notable change in maternal blood pressure. Meanwhile, a pilot study of remotely delivered mindfulness training among a small sample of pregnant women with a history of hypertensive disorders reported benefits for reduction of systolic and diastolic blood pressure (Bublitz et al., 2023). However, a risk of bias in the results of that study was noted due to failure to statistically compare key characteristics between intervention and control groups at baseline which could indicate unmeasured confounding in the analysis. Similarly, the apparent positive results on post-stressor blood pressure values reported by Muthukrishnan et al. (2016) following their 5-week mindful meditation program should be interpreted with caution given the high risk of bias in that study due to failure to report various methodological procedures as well as participant retention and adherence rates. Only two observational studies were identified that reported on blood pressure as an outcome variable but neither found any significant association with maternal trait mindfulness (Braeken et al., 2017; Mennitto et al., 2021), although the credibility of one of these studies is questionable due to major flaws and a high risk of bias (Mennitto et al., 2021).

### Comparison with Existing Studies from Pregnant and Non-pregnant Populations

Evidence from a growing body of intervention trials in non-pregnant adults demonstrates that increasing psychological well-being has the capacity to causally improve metabolic health outcomes. The most commonly used psychological well-being interventions have involved mindfulness and yoga practice and provide evidence for changes across multiple indicators of metabolic health. Specifically, several systematic reviews have concluded that psychological well-being interventions, primarily yoga based, can improve measures of glycemic control, including fasting and post-prandial glucose, HbA1c, and measures of adiposity and obesity risk (Chew et al., 2017; Jayawardena et al., 2018; Pascoe et al., 2017; Ramamoorthi et al., 2019; Thind et al., 2017). A further two systematic reviews report beneficial effects of mindfulness-based interventions on weight loss and obesity-related eating behaviors (Carriere et al., 2018; Dunn et al., 2018). In addition, four systematic reviews have interrogated the effects of mindfulness meditation or yoga practice on changes to the immune system, and all concluded there were reductions in proinflammatory processes, with evidence for cell-mediated immunity (Black & Slavich, 2016; Djalilova et al., 2019; Falkenberg et al., 2018; Moraes et al., 2018) and dose–response improvements (Black & Slavich, 2016; Djalilova et al., 2019).

These studies provide biological plausibility and support the translation of mindfulness-based interventions for the improvement of maternal cardiometabolic health in pregnancy. This is a growing field and, thus far in the context of pregnancy, systematic reviews have focused on the effects of yoga and mindfulness interventions upon broad categories of maternal psychological health, including quality of life, self-efficacy, perceived stress, anxiety, and depression scores. Of the five systematic reviews that investigated the effects of yoga (Curtis et al., 2012; Kawanishi et al., 2015; Kwon et al., 2020; Mooventhan, 2019; Sheffield & Woods-Giscombe, 2016) and the three that investigated mindfulness interventions (Dhillon et al., 2017; Hall et al., 2016; Lever Taylor et al., 2016), all argue in favor of positive intervention effectiveness for psychological health outcomes. However, there are broad concerns regarding the limited number of RCTs, particularly with active controls, inconsistency across the types of intervention practices used, and a lack of understanding regarding which components of the intervention are necessary to drive the perceived improvements in psychological health, all of which are shared concerns with the studies included in this integrative review.

### Limitations and Future Research

This integrative review is the first to synthesize the literature on mindfulness during pregnancy in relation to cardiometabolic health outcomes, thereby expanding our current knowledge of potential benefits of mindfulness beyond the psychological realm alone. By including diverse study designs in our review, we have captured broad perspectives about the potential relevancy of considering maternal trait mindfulness in relation to cardiometabolic health in pregnancy, as well as the acceptability and efficacy of prenatal mindfulness interventions for supporting maternal cardiometabolic health. Our rigorous literature search was conducted in partnership with a research librarian to ensure accuracy, and incorporated search terms that spanned four domains of cardiometabolic health (weight gain, glycemic control, blood pressure, inflammation) to facilitate broad capture of studies that addressed physiological health measures with established links to maternal and offspring cardiometabolic health during pregnancy and beyond. Furthermore, we conducted an extensive quality assessment of included studies to help guide the interpretation and reliability of results reported.

We note two limitations of this review. Firstly, although our search was not restricted by country or year of publication, we did limit the search to articles published in the English language which could have resulted in exclusion of other potentially relevant studies. Secondly, we restricted our eligibility to studies that reported maternal cardiometabolic health outcomes during pregnancy, which may have missed studies that demonstrate postpartum maternal or offspring cardiometabolic health benefits associated with mindfulness traits assessed, or interventions delivered, during the prenatal period.

Given the high rates of maternal morbidity and mortality in the United States and worldwide (Centers for Disease Control & Prevention, 2020, 2021; Kotit & Yacoub, 2021), which are often related to poor cardiometabolic health in the pre- and perinatal periods (Marschner et al., 2022), there is a critical need to consider all avenues for prevention and reduction of adverse pregnancy-related outcomes through safe, accessible, and patient-centered adjunctive approaches to traditional medical management. The evidence presented in this integrative review highlights prenatal mindfulness as a potentially relevant target for further research in this regard. However, the literature published on this topic to date is heterogeneous with respect to study design, interventions, and outcome measures and the majority of identified studies suffer from moderate to high risk of bias. Thus, the largely null results reported in this review should not discourage researchers from conducting further studies of mindfulness and cardiometabolic health in pregnant populations as there is a need for more rigorous data to determine the potential pathways of associations and efficacy of interventions for improving maternal physiological health outcomes. Moreover, since the emotional and mental well-being benefits of mindfulness practice during pregnancy have been repeatedly shown in prior studies (Dhillon et al., 2017; Hall et al., 2016; Lever Taylor et al., 2016; Lucena et al., 2020), pregnant participants of future research trials that aim to examine the impact on gestational cardiometabolic health outcomes can, at the very least, expect to find mental health benefits from establishing a regular mindfulness practice.

Future prenatal mindfulness intervention trials should aim for randomized designs that are adequately powered to test the impact on pre-defined cardiometabolic health outcome(s). They should consider an active control group, such as group classes on general pregnancy well-being, rather than standard prenatal care, which may provide a more rigorous comparison against the effects of mindfulness-based interventions. Participant retention, handling and reporting of missing data, and conducting intention-to-treat analyses that appropriately account for missing data are currently major weaknesses that must be addressed to elevate the quality of evidence in this field. More complex study designs may also be required to tease apart the effects of mindfulness intervention components from more traditional behavior change approaches that target maternal cardiometabolic health (e.g., nutrition counseling, exercise), and determine whether cardiometabolic health benefits in pregnancy can be augmented when these intervention components are administered in combination. A significant knowledge gap remains with respect to the effects of prenatal mindfulness-based interventions on maternal markers of inflammation, as no studies reporting on inflammatory markers, even as secondary outcome measures, were identified by this review. Yet, immune/inflammatory-related pathways represent a plausible mechanism by which mindfulness interventions may exert beneficial effects on weight gain, glycemic control, and blood pressure, as well as numerous other potential adverse pregnancy outcomes. There may also be other physiological markers of interest, such as maternal resting heart rate, that could benefit from prenatal mindfulness interventions and represent markers of downstream maternal and offspring cardiometabolic health outcomes. For example, low maternal heart rate has been associated with lower birthweight babies (Odendaal et al., 2019), while high resting heart rate has been linked to elevated risk of GDM (Qiu et al., 2022).

Observational studies have potential to generate important data to inform the need for and direction of intervention trials. Yet, we identified less observational compared to intervention studies in this review and two of the included observational studies were deemed to have critical flaws that limit the interpretability and reliability of the data reported. Moreover, none of the observational studies examined inflammatory markers as outcome measures, representing a missed opportunity to capture data that could inform the underlying mechanisms by which mindfulness practice may exert positive cardiometabolic effects. We recommend that future observational studies assess maternal trait mindfulness and mindful eating behavior in pregnancy and examine their associations with objectively measured markers of cardiometabolic health that span the domains of weight gain, glycemic control, blood pressure, and inflammation. Consistency in measures used to assess mindfulness, as well as in gestational timing of assessments, will assist with replication across studies.

Qualitative studies also provide valuable insight to participants’ attitudes toward and experiences of mindfulness as a tool to support their physiological health, in addition to the established psychological health benefits. The only qualitative studies that met eligibility criteria for this review focused on mindfulness in relation to GWG. Given the known associations between psychological stress and cardiometabolic risk factors including poor glycemic control, elevated blood pressure, and inflammation, and the potential for mindfulness practice to improve resiliency and psychological well-being, there is a need to explore whether pregnant individuals might also be receptive to mindfulness interventions with the goal of improving a broader spectrum of physiological health markers beyond GWG alone. Further, given that most studies of mindfulness interventions conducted in Western countries to date primarily target non-Hispanic White and highly educated populations (Cramer et al., 2016; Khoury et al., 2015), more qualitative data are required to elucidate how future interventions in this field can be more inclusive of and appropriately tailored toward diverse racial and ethnic groups and those from lower socioeconomic status backgrounds. This is especially important given that individuals from racial and ethnic minority groups are more likely to be affected by poor cardiometabolic health outside of and during pregnancy compared to non-Hispanic White individuals (Akam et al., 2022; Bentley-Lewis et al., 2014; Hedderson et al., 2010; Ross et al., 2020). Indeed, the included qualitative study by Thomas et al. (2014) found that low-income, predominantly Hispanic pregnant women were interested in a pregnancy-tailored mindfulness intervention, but it remains to be determined if other minority groups in the US, or low-income people from different geographical locations, also share these beliefs.

This integrative review identified mixed evidence, primarily of fair-to-poor quality, for potential benefits of mindfulness practices to support cardiometabolic health in pregnancy. More rigorous evidence exists from non-pregnancy studies regarding beneficial effects of mindfulness-based or yoga interventions on inflammatory processes, glycemic control, and adiposity. Thus, further rigorous studies are required to understand whether mindfulness is an efficacious approach, either alone or in combination with other lifestyle modalities, to improve gestational cardiometabolic health outcomes, as well as potential downstream physiologic health benefits for the offspring and for the mothers postpartum.

## Supplementary Material

Supply Information_Search criteria

Suppl Table S1

Suppl Table S2

## Figures and Tables

**Table 1:** Characteristics of included quantitative studies

*Intervention studies*					
Author, year	Study design	Location	Population	Intervention (I)/control (C)	Cardiometabolic Outcome(s)
Bublitz et al., 2023	RCT; 2 parallel arms	USA	N=29, singleton pregnancy, age ≥18y, with a history of hypertensive disorder, absence of severe depressive symptoms; enrolled at ≤20 weeks	I: Eight weekly 30-minute mindfulness training sessions delivered by phone, emphasizing awareness of breath and body scan techniques from the standard MBSR curriculum. Home practice of these techniques also prescribed. C: Standard prenatal medical care including medical treatment of hypertension as needed; weekly phone call check-ins asking how they were feeling and attendance at routine prenatal care appointments	Hypertensive disorders of pregnancy; blood pressure
Crovetto et al., 2021	RCT; 3 parallel arms	Spain	N=1221, singleton pregnancy, age ≥18y, at high risk for delivering a small-for-gestational age baby; enrolled at 19–23 weeks	I: Monthly dietitian consults on the Mediterranean diet and free provision of olive oil and walnuts I: Eight-week standardized MBSR program, home practices strongly encouraged during and after the course, additional mindfulness/yoga sessions available after course completion. C: Standard prenatal medical care	Preeclampsia, GDM, gestational hypertension, blood pressure
Epel et al., 2019	Quasi-experimental	USA	N=215, singleton pregnancy, low-income, pregravid BMI 25–40, age 18–45y, no diabetes; enrolled between 12–19 weeks (Intervention group), and up to 23 weeks (control group)	I: Mindful Moms Training (MMT): 8 weekly 2-hr group classes plus 2 telephone sessions beginning in early 2^nd^ trimester. Classes included didactic and experiential components, covering mindfulness based stress reduction, mindful eating and healthy nutrition advice as well as prescribed homework on these activities. C: Convenience sample receiving standard prenatal medical care	GWG and rate of excess GWG; glucose tolerance
Muthukrishnan et al., 2016	RCT; 2 parallel arms	India	N=74, singleton pregnancy without complications (including obesity or psychiatric problems), age ≥18y; enrolled at 12 weeks	I: Five week mindful meditation program adapted from MBSR; 2 sessions per week plus 30 mins daily home practice. C: Standard prenatal medical care	Blood pressure at rest and blood pressure increase in response to cold pressor and mental arithmetic tests
Opie et al., 2018	Quasi-experimental	Australia	N=217, singleton pregnancy, BMI 30–35, age ≥18y, uncomplicated pregnancy; enrolled at >20 weeks	I: One dietitian consult focused on healthy eating and achieving adequate GWG, plus monthly follow-up calls to review and support achievement of personalized nutrition goals; mindfulness approaches to eating were incorporated into dietary consults in a non-standardized manner. C: Convenience sample receiving standard prenatal medical care	GDM, GWG
Redman et al., 2017	RCT; 3 parallel arms	USA	N=54, singleton pregnancy, pregravid BMI ≥25, no diabetes, no history or current episode of major depression or psychotic disorder, aged 18–40y; enrolled at 13 weeks	I: SmartMoms: 18 lessons on diet and behavior modification strategies from 13 weeks gestation until delivery, delivered as a combination of group and individualized sessions. Intervention group randomized to receive the intervention either in-person or remotely through smartphone application plus contact through email, phone calls and text messages, with identical content. Mindfulness stress reduction and mindful eating guidance included during 2 group and 2 individual sessions, remaining sessions counselled on diet, physical activity, and monitoring GWG. C: Standard prenatal medical care	GWG and rate of excess GWG
Youngwanichsetha et al., 2014	RCT; 2 parallel arms	Thailand	N=170 pregnant individuals with GDM, fasting and postprandial blood glucose <105 and <120 mg/dl respectively, not receiving insulin therapy, no other pregnancy complications (e.g. hypertension, preeclampsia, preterm labor), mean age 32y, multiple pregnancy eligibility not defined; enrolled 24–30 weeks	I: Mindful eating and yoga: two 50min training sessions using videos and practice manuals, followed by 8 weeks of at-home practice for 5 days/week. Yoga comprised of a 15–20min practice of 9 pregnancy-modified postures repeated 10 times. Mindful eating comprised of setting blood glucose goals, modifying carbohydrate portion and selecting low GI foods, awareness while eating and eating slowly for 35–40mins. C: standard prenatal care for women with GDM	Fasting and postprandial glucose concentrations; HbA1c
*Observational studies*					
Author, year	Study design	Location	Population	Mindfulness-related Exposure	Cardiometabolic Outcome(s)
Braeken et al., 2017	Prospective cohort	Netherlands	N=156, low-risk pregnancies, mean pregravid BMI=24; enrolled at 8–14 weeks	Maternal trait mindfulness, assessed by the Freiberg Mindfulness Inventory short form (14 item)	Systolic and diastolic blood pressure measured in the first and third trimester
Headen et al., 2019	Retrospective, observational	USA	N=207, singleton pregnancy, low-income, pregravid BMI 25–40, age 18–45y, no diabetes; enrolled between 12–23 weeks	Mindful Moms Training as described under the intervention study by Epel et al. This study evaluated the moderating effects of participant neighborhood typology on the efficacy of the mindfulness intervention	Rate of excess GWG; glucose tolerance
Lindsay et al., 2021	Prospective cohort	USA	N=46, singleton pregnancy, age 18–40y, pregravid BMI ≥30, without pre-existing conditions such as hypertension, diabetes or psychological disorders; enrolled at <15 weeks	Mindful eating assessed by the Mindful Eating Questionnaire in early and late pregnancy; mean pregnancy total score and subscale scores were used in the analysis	Rate of GWG/week, rate of adiposity gain/week, homeostasis model assessment of insulin resistance measured at 35–37 weeks gestation
Matthews et al., 2018	Cross-sectional survey	USA	N=1073 pregnant people, age ≥18y,	Maternal trait mindfulness, assessed by the Mindful Attention and Awareness Scale (15 item)	GWG in each trimester
Mennitto et al., 2021	Cross-sectional	Canada	N=510, singleton pregnancy, age ≥18y; enrolled at <20 weeks	Maternal trait mindfulness, assessed by the Mindful Attention and Awareness Scale (15 item) in the first or early second trimester	Maternal self-reported diagnosis of GDM or high blood pressure in pregnancy

BMI, body mass index; GDM, gestational diabetes mellitus; GWG, gestational weight gain; MBSR, mindfulness-based stress reduction; RCT, randomized controlled trial

**Table 2:** Results of included quantitative studies that pertain to cardiometabolic health outcomes

Author, year	Covariates considered	Weight gain related outcomes	Glycemic related outcomes	Blood pressure related outcomes
***Intervention studies***				
Bublitz et al., 2023	Baseline blood pressure values	Not assessed	Not assessed	Systolic blood pressure (mmHg) at follow-up: Intervention (mean±SD: 114±6) significantly lower than control (119±4), p=0.026. Diastolic blood pressure (mmHg) at follow-up: Intervention (mean±SD: 71±6) significantly lower than control (79±6), p=0.004.
Crovetto et al., 2021	Baseline mean blood pressure adjusted in the analysis of differences in follow-up blood pressure readings across groups	Not assessed	GDM: rates by group: Med diet 12.2% (38/392), stress reduction 9.7% (38/391), usual care 7.5% (30/401); sig difference only found between Med diet and usual care groups (p=0.03)	Preeclampsia: Rates by group: Med diet 5.6% (22/386), stress reduction 6.2% (24/381), usual care 9.3% (37/389). Risk difference (95% CI) between stress reduction and usual care group not significant [−3.1 (−6.8 to 0.6), p=0.11]. Gestational hypertension: Rates by group: Med diet 2% (8/392), stress reduction 2.8% (11/391), usual care 2.3% (9/401); no significant differences between groups. Blood pressure: Mean±SD values (mmHg) at final prenatal assessment: Med diet 84.1±0.4, stress reduction 83.5±0.4, usual care 82.7±0.4; p (ANOVA)=0.04, sig difference only between Med diet and usual care.
Epel et al., 2019	Age, pre-pregnancy BMI, parity	Proportion exceeding GWG guideline: intervention 67%, 64/95 vs control: 69%, 62/90; p=0.44. Proportion below IOM guideline: intervention 11%, 10/95 vs control 20%, 19/90; p=0.026. Total GWG: intervention gained 0.05 kg more than control, p=0.98	OGTT 1hr glucose result: N=141 subset at 24 weeks (mean ±SD): intervention 111.8±27.7 vs control 100.3±23.3 mg/dL; p=0.009; Impaired glucose tolerance (1hr glucose >130 mg/dL): intervention 8.3%, 6/72 vs control 20.6%, 14/68; OR=0.34, p= 0.04.	Not assessed
Muthukrishnan et al., 2016	None	Not assessed	Not assessed	Systolic blood pressure (mmHg) at follow-up: no difference between intervention and control groups at rest (mean±SD: 109.2±3.8 vs 124.7±5.6; p=0.52), significantly smaller increase in the intervention group vs control in response to the cold pressure test (9.7±1.8 vs 13.4±2.4, p<0.001) and in response to a mental arithmetic test (9.0±2.2 vs 13.4±2.2). Diastolic blood pressure (mmHg) at follow-up: no difference between intervention and control groups at rest (mean±SD: 69.1±2.2 vs 69.1±2.2 p=1.00), significantly smaller increase in the intervention group vs control in response to the cold pressure test (4.2±1.0 vs 7.5±1.4, p<0.001) but not in response to a mental arithmetic test (5.2±1.5 vs 4.4±1.3, p=0.056).
Opie et al., 2018	BMI and ethnicity	Total GWG: Intervention 10.0±4.8 kg vs control 9.7±5.5 kg, p>0.05. (note: GWG data only available for 89% of intervention group and 46% of control group)	GDM: Unadjusted model: intervention 6.5% (6/92) vs control 19.3% (23/119). In the adjusted model, group allocation was not associated with GDM diagnosis (OR=0.49, 95%CI 0.16–1.55)	Not assessed
Redman et al., 2017	None	Proportion exceeding GWG guideline: both in-person (56%, 10/18) and remote (58%, 11/19) interventions significantly lower than control (85%, 11/13); p=0.03 and p=0.04 respectively.	Not assessed	Not assessed
Youngwanichsetha et al., 2014	None	Not assessed	Fasting glucose: (mean±SD): intervention 83.39±17.69 vs control 85.85±17.94 mg/dl, p=0.012. 2hr post-prandial glucose: (mean±SD): intervention 105.67±12.93 vs control 112.36±13.15 mg/dl, p=0.001. HbA1c: (mean±SD): intervention 5.23%±0.72 vs control 5.68%±0.68 mg/dl, p=0.038.	Not assessed
***Observational studies***				
Braeken et al., 2017	Emotional distress, maternal age, BMI, education level	Not assessed	Not assessed	Systolic blood pressure: Values in first trimester, nor change between first and third trimesters were associated with trait mindfulness (all p>0.05). Diastolic blood pressure: Values in first trimester, nor change between first and third trimesters were associated with trait mindfulness (all p>0.05).
Headen et al., 2019		Rate of excess GWG: Neighborhood typology did not modify the effects of the mindfulness intervention compared to control (data not available).	Glucose result from 1hr glucose challenge test: neighborhood type moderated the effectiveness of the mindfulness intervention such that significantly lower glucose results were only found among those living in wealthy neighborhoods with high resource access [mean difference (95% CI): −21.0 (−36.8, −5.1) mg/dl] or those in poor neighborhoods with moderate resource access [−36.3 (−55.4, −17.3) mg/dl] in the intervention group compared to control. Null intervention effects among those living in middle income, low resource access or poor, moderate resource access neighborhoods.	Not assessed
Lindsay et al., 2021	Maternal age, pregravid BMI, race, parity, diet quality score	Rate of GWG: No association with total mindful eating score (beta=−0.13, p=0.24). Inverse association with distracted eating subscale score (i.e. less tendency for distracted eating associated with lower rate of GWG; beta=−0.13, p=0.03). Rate of fat mass gain: No association with total mindful eating score (beta=−1.19, p=0.44). Inverse association with distracted eating subscale score (i.e. less tendency for distracted eating associated with lower rate of fat mass gain; beta=−2.07, p=0.02).	Insulin resistance (HOMA-IR): Inverse association with total mindful eating score in adjusted model (beta=−0.28, p=0.03). No association with any of the mindful eating subscales (all p>0.05).	Not assessed
Matthews et al., 2018	Age, smoking, alcohol use, pre-pregnancy BMI, physical activity, income and education.	GWG by trimester: Trait mindfulness was significantly inversely correlated with GWG in the first (r=−0.66, p<0.01) and second (r=−0.13, p<0.01) trimesters but not in the third trimester (r=−0.04, p>0.05). In adjusted models, mindfulness remained significantly associated with GWG only in the first trimester (beta=−3.82, p<0.001).	Not assessed	Not assessed
Mennitto et al., 2021	None	Not assessed	Incidence of GDM: No association with trait mindfulness (data not available).	Incidence of hypertension: No association with trait mindfulness (data not available).

## Data Availability

No original data were generated in this research. All data are presented in the paper and Supplementary Material.
