# Supplementary material for: Mindfulness and Cardiometabolic Health During Pregnancy: An Integrative Review
Source: Mindfulness (N Y). Author manuscript; Available in PMC 2025 Jan 17. (PMC11741670; doi:10.1007/s12671-024-02337-2)
Supplement: Supply Information_Search criteria [file NIHMS2007616-supplement-Supply_Information_Search_criteria.pdf]

Article Title: Mindfulness and cardiometabolic health during pregnancy: An integrative review
Journal: Mindfulness
Authors: Karen L. Lindsay*, Yuqing Guo, Lauren E. Gyllenhammer
***Corresponding author affiliation:** School of Medicine, Department of Pediatrics, University of California, Irvine. kllindsa@hs.uci.edu

Literature Search Strategy

("Pregnancy"[Mesh] OR pregnan* OR "Prenatal Care"[Mesh] OR prenatal OR antenatal OR maternal) AND ("Mindfulness"[Mesh] OR mindful*) AND ("Gestational Weight Gain"[Mesh] OR "weight gain" OR "Blood Glucose"[Mesh] OR glucose OR "Glycemic Control"[Mesh] OR glycemia OR HbA1c OR "Hb A1c" OR "Insulin"[Mesh] OR insulin OR pregnancy-induced diabetes OR "Diabetes, Gestational"[Mesh] OR gestational diabetes OR "beta-cell function" OR "beta-cell dysfunction" OR hypertension OR "Hypertension, Pregnancy-Induced"[Mesh] OR pregnancy induced hypertension OR gestational hypertension OR pregnancy transient hypertension OR preeclampsia OR "Pre-Eclampsia"[Mesh] OR "pre eclampsia" OR "pregnancy toxemia*" OR "blood pressure" OR "Blood Pressure"[Mesh] OR inflammat* OR "Inflammation"[Mesh] OR cytokine OR "Cytokines"[Mesh] OR interleukin)
Filters: English
