# Supplementary material for: Mindfulness and Cardiometabolic Health During Pregnancy: An Integrative Review
Source: Mindfulness (N Y). Author manuscript; Available in PMC 2025 Jan 17. (PMC11741670; doi:10.1007/s12671-024-02337-2)
Supplement: Suppl Table S1 [file NIHMS2007616-supplement-Suppl_Table_S1.pdf]

Article Title: Mindfulness and cardiometabolic health during pregnancy: An integrative review
Journal: Mindfulness
Authors: Karen L. Lindsay*, Yuqing Guo, Lauren E. Gyllenhammer
***Corresponding author affiliation:** School of Medicine, Department of Pediatrics, University of California, Irvine.
kllindsa@hs.uci.edu

Table S1-A. Quality Assessment of Controlled Intervention Studies: Randomized Control Trials and Quasi-Experimental

Criteria	Study						
	Bublitz, 2023	Crovetto, 2021	Epel, 2019	Muthukrishnan, 2016	Opie, 2016	Redman, 2017	Youngwanichsetha, 2014
1. Was the study described as randomized, a randomized trial, a randomized clinical trial, or an RCT?	Y	Y	N- Quasi	Y	N- Quasi	Y	Y
2. Was the method of randomization adequate (i.e., use of randomly generated assignment)?	Y	Y	NA	CD	NA	Y	CD
3. Was the treatment allocation concealed (so that assignments could not be predicted)?	Y	Y	NA	NR	NA	Y	Y
4. Were study participants and providers blinded to treatment group assignment?	NA	NA	NA	NA	NA	NA	NA
5. Were the people assessing the outcomes blinded to the participants' group assignments?	NR	Y	NR	Y	NR	Y	NR
6. Were the groups similar at baseline on important characteristics that could affect outcomes (e.g., demographics, risk factors, co-morbid conditions)?	CD	Y	N	Y	N	Y	Y
7. Was the overall drop-out rate from the study at endpoint 20% or lower of the number allocated to treatment?	Y	Y	Y	CD	Y	NR	Y
[illegible] groups) at endpoint 15 percentage points or lower?	Y	Y	Y	CD	N	NR	Y
9. Was there high adherence to the intervention protocols for each treatment group?	Y	N	Y	CD	Y	N	Y
10. Were other interventions avoided or similar in the groups (e.g., similar background treatments)?	Y	Y	Y	NR	NR	NR	NR
11. Were outcomes assessed using valid and reliable measures, implemented consistently across all study participants?	Y	Y	Y	N	Y	Y	N
12. Did the authors report that the sample size was sufficiently large to be able to detect a difference in the main outcome between groups with at least 80% power?	N	Y	Y	CD	Y	NR	Y

13. Were outcomes reported or subgroups analyzed prespecified (i.e., identified before analyses were conducted)?	**N**	**Y**	**Y**	**CD**	**NR**	**Y**	**CD**
14. Were all randomized participants analyzed in the group to which they were originally assigned, i.e., did they use an intention-to-treat analysis?	**Y**	**Y**	**Y**	**CD**	**Y**	**Y**	**N**
**Overall Quality Rating**	**Fair**	**Good**	**Fair**	**Poor**	**Fair**	**Fair**	**Poor**
**Key Score Comment for Fair or Poor Ratings**	Outcome measurement calculation not pre-specified, no stastistical comparison of baseline differences in outcome and other patient sociodemographic variables		Described as ITT analysis, but missing outcome data wasn't accounted for (missing psychological and GWG outcome data in 23% and 11%). Not a true ITT analysis. Baseline differences in groups.	Cannot determine for most questions and cannot determine ITT analysis	Described as ITT analysis, but missing outcome data wasn't accounted for (missing outcome data in 17% due to hospital transfer)). Not a true ITT analysis. Baseline differences in groups.	Limited by the differential adherence of the treatment groups (77% vs 61%) and the non-reporting of drop-out—limits the ability to determine if a "true" ITT was conducted	No description/ confirmation of ITT analysis, outcome measurement not described for post-prandial glucose testing, and method of randomization not well described.

**Legend: Y=yes; N=no; CD= cannot determine; NR= not reported; NA= not applicable; ITT=intent-to-treat*

Table S1-B. Quality Assessment of Observational Cohort and Cross-sectional Studies

Criteria	Study				
	Braeken, 2017	Headen, 2019	Lindsay, 2021	Matthews , 2018	Mennitto, 2021
1. Was the research question or objective in this paper clearly stated?	Y	Y	Y	Y	Y
2. Was the study population clearly specified and defined?	N	Y	Y	Y	Y
3. Was the participation rate of eligible persons at least 50%?	NR	Y	NR	NR	NR
4. Were all the subjects selected or recruited from the same or similar populations (including the same time period)? Were inclusion and exclusion criteria for being in the study prespecified and applied uniformly to all participants?	CD	Y	Y	Y	Y
5. Was a sample size justification, power description, or variance and effect estimates provided?	N	N	N	N	N
6. For the analyses in this paper, were the exposure(s) of interest measured prior to the outcome(s) being measured?	N	N	N	N	Y
7. Was the timeframe sufficient so that one could reasonably expect to see an association between exposure and outcome if it existed?	Y	Y	Y	Y	Y
8. For exposures that can vary in amount or level, did the study examine different levels of the exposure as related to the outcome (e.g., categories of exposure, or exposure measured as continuous variable)?	Y	Y	Y	Y	Y
9. Were the exposure measures (independent variables) clearly defined, valid, reliable, and implemented consistently across all study participants?	Y	Y	Y	Y	Y
10. Was the exposure(s) assessed more than once over time?	N	N	N	N	N
11. Were the outcome measures (dependent variables) clearly defined, valid, reliable, and implemented consistently across all study participants?	Y	Y	Y	N	N
12. Were the outcome assessors blinded to the exposure status of participants?	NR	NR	NR	N	N
13. Was loss to follow-up after baseline 20% or less?	Y	Y	N	NA	N
14. Were key potential confounding variables measured and adjusted statistically for their impact on the relationship between exposure(s) and outcome(s)?	Y	Y	N	Y	N
**Overall Quality Rating**	Fair	Good	Fair	Poor	Poor

**Key Score Comment for Fair or Poor Ratings**		Study population not clearly defined and unclear if outcome assessment was collected in a blinded manner		Secondary analysis with only 64% of original cohort included and not discussed (could introduce bias), and key potential confounds were not included in the analyses (e.g., socioeconomic status (income/education) related psychological constructs)	Critical issue of poor outcome measurement description	No discussion or control for potential confounders (important source of bias in cross-sectional studies); maternal health outcomes (high blood pressure and GDM) measured as self report, which is not ideal outcome measurement, could introduce bias; large loss from baseline (only 66.8% complete all 3)

**Legend:* Y=yes; N=no; CD= cannot determine; NR= not reported; NA= not applicable; ITT=intent-to-treat*
