# Supplementary material for: Mindfulness and Cardiometabolic Health During Pregnancy: An Integrative Review
Source: Mindfulness (N Y). Author manuscript; Available in PMC 2025 Jan 17. (PMC11741670; doi:10.1007/s12671-024-02337-2)
Supplement: Suppl Table S2 [file NIHMS2007616-supplement-Suppl_Table_S2.pdf]

Article Title: Mindfulness and cardiometabolic health during pregnancy: An integrative review
Journal: Mindfulness
Authors: Karen L. Lindsay*, Yuqing Guo, Lauren E. Gyllenhammer
***Corresponding author affiliation:** School of Medicine, Department of Pediatrics, University of California, Irvine. kllindsa@hs.uci.edu

Table S2: Characteristics of included quantitative studies

Intervention studies					
**Author, year**	**Study design**	**Location**	**Population**	**Intervention (I)/control (C)**	**Cardiometabolic Outcome(s)**
Bublitz et al., 2023	RCT; 2 parallel arms	USA	N=29, singleton pregnancy, age ≥18y, with a history of hypertensive disorder, absence of severe depressive symptoms; enrolled at ≤20 weeks	I: Eight weekly 30-minute mindfulness training sessions delivered by phone, emphasizing awareness of breath and body scan techniques from the standard MBSR curriculum. Home practice of these techniques also prescribed.<br>C: Standard prenatal medical care including medical treatment of hypertension as needed; weekly phone call check-ins asking how they were feeling and attendance at routine prenatal care appointments	Hypertensive disorders of pregnancy; blood pressure
Crovetto et al., 2021	RCT; 3 parallel arms	Spain	N=1221, singleton pregnancy, age ≥18y, at high risk for delivering a small-for-gestational age baby; enrolled at 19-23 weeks	I: Monthly dietitian consults on the Mediterranean diet and free provision of olive oil and walnuts<br>I: Eight-week standardized MBSR program, home practices strongly encouraged during and after the course, additional mindfulness/yoga sessions available after course completion.<br>C: Standard prenatal medical care	Preeclampsia, GDM, gestational hypertension, blood pressure
Epel et al., 2019	Quasi-experimental	USA	N=215, singleton pregnancy, low-income, pregravid BMI 25-40, age 18-45y, no diabetes; enrolled between 12-19 weeks (Intervention group), and up to 23 weeks (control group)	I: Mindful Moms Training (MMT): 8 weekly 2-hr group classes plus 2 telephone sessions beginning in early 2nd trimester. Classes included didactic and experiential components, covering mindfulness based stress reduction, mindful eating and healthy nutrition advice as well as prescribed homework on these activities.	GWG and rate of excess GWG; glucose tolerance

				C: Convenience sample receiving standard prenatal medical care	
Muthukrishnan et al., 2016	RCT; 2 parallel arms	India	N=74, singleton pregnancy without complications (including obesity or psychiatric problems), age ≥18y; enrolled at 12 weeks	I: Five week mindful meditation program adapted from MBSR; 2 sessions per week plus 30 mins daily home practice.<br>C: Standard prenatal medical care	Blood pressure at rest and blood pressure increase in response to cold pressor and mental arithmetic tests
Opie et al., 2018	Quasi-experimental	Australia	N=217, singleton pregnancy, BMI 30-35, age ≥18y, uncomplicated pregnancy; enrolled at >20 weeks	I: One dietitian consult focused on healthy eating and achieving adequate GWG, plus monthly follow-up calls to review and support achievement of personalized nutrition goals; mindfulness approaches to eating were incorporated into dietary consults in a non-standardized manner.<br>C: Convenience sample receiving standard prenatal medical care	GDM, GWG
Redman et al., 2017	RCT; 3 parallel arms	USA	N=54, singleton pregnancy, pregravid BMI ≥25, no diabetes, no history or current episode of major depression or psychotic disorder, aged 18-40y; enrolled at 13 weeks	I: SmartMoms: 18 lessons on diet and behavior modification strategies from 13 weeks gestation until delivery, delivered as a combination of group and individualized sessions. Intervention group randomized to receive the intervention either in-person or remotely through smartphone application plus contact through email, phone calls and text messages, with identical content. Mindfulness stress reduction and mindful eating guidance included during 2 group and 2 individual sessions, remaining sessions counselled on diet, physical activity, and monitoring GWG.<br>C: Standard prenatal medical care	GWG and rate of excess GWG
Youngwanichsetha et al., 2014	RCT; 2 parallel arms	Thailand	N=170 pregnant individuals with GDM, fasting and postprandial blood glucose <105 and <120 mg/dl respectively, not receiving insulin therapy, no other pregnancy complications	I: Mindful eating and yoga: two 50min training sessions using videos and practice manuals, followed by 8 weeks of at-home practice for 5 days/week. Yoga comprised of a 15-20min practice of 9 pregnancy-modified postures repeated 10 times. Mindful eating comprised of setting blood glucose goals, modifying carbohydrate portion and selecting low GI foods,	Fasting and postprandial glucose concentrations; HbA1c

			(e.g. hypertension, preeclampsia, preterm labor), mean age 32y, multiple pregnancy eligibility not defined; enrolled 24-30 weeks	awareness while eating and eating slowly for 35-40mins.<br>C: standard prenatal care for women with GDM	
Observational studies					
**Author, year**	**Study design**	**Location**	**Population**	**Mindfulness-related Exposure**	**Cardiometabolic Outcome(s)**
Braeken et al., 2017	Prospective cohort	Netherlands	N=156, low-risk pregnancies, mean pregravid BMI=24; enrolled at 8-14 weeks	Maternal trait mindfulness, assessed by the Freiberg Mindfulness Inventory short form (14 item)	Systolic and diastolic blood pressure measured in the first and third trimester
Headen et al., 2019	Retrospective, observational	USA	N=207, singleton pregnancy, low-income, pregravid BMI 25-40, age 18-45y, no diabetes; enrolled between 12-23 weeks	Mindful Moms Training as described under the intervention study by Epel et al. This study evaluated the moderating effects of participant neighborhood typology on the efficacy of the mindfulness intervention	Rate of excess GWG; glucose tolerance
Lindsay et al., 2021	Prospective cohort	USA	N=46, singleton pregnancy, age 18-40y, pregravid BMI ≥30, without pre-existing conditions such as hypertension, diabetes or psychological disorders; enrolled at <15 weeks	Mindful eating assessed by the Mindful Eating Questionnaire in early and late pregnancy; mean pregnancy total score and subscale scores were used in the analysis	Rate of GWG/week, rate of adiposity gain/week, homeostasis model assessment of insulin resistance measured at 35-37 weeks gestation
Matthews et al., 2018	Cross-sectional survey	USA	N=1073 pregnant people, age ≥18y,	Maternal trait mindfulness, assessed by the Mindful Attention and Awareness Scale (15 item)	GWG in each trimester
Mennitto et al., 2021	Cross-sectional	Canada	N=510, singleton pregnancy, age ≥18y; enrolled at <20 weeks	Maternal trait mindfulness, assessed by the Mindful Attention and Awareness Scale (15 item) in the first or early second trimester	Maternal self-reported diagnosis of GDM or high blood pressure in pregnancy

BMI, body mass index; GDM, gestational diabetes mellitus; GWG, gestational weight gain; MBSR, mindfulness-based stress reduction; RCT, randomized controlled trial
